# Research hotspots and trend of the heart-brain axis by MRI: a bibliometric analysis

**DOI:** 10.3389/fcvm.2026.1825341

**Published:** 2026-07-07

**Authors:** Haoran Wang, Yang Jia, Yi Liao, Linjun Xie, Wenfeng Jin, Qianyu Peng, Huayan Xu, Rong Xu, Yingkun Guo

**Affiliations:** 1 Department of Radiology, Sichuan University West China Second University Hospital, Chengdu, China; 2 Key Laboratory of Birth Defects and Related Diseases of Women and Children, Sichuan University, Chengdu, China

**Keywords:** bibliometric analysis, heart-brain axis, MRI, neuroimaging, research trends

## Abstract

**Background:**

The “Heart–Brain Axis” (HBA) represents an emerging interdisciplinary domain in which cardiac dysfunction is increasingly recognized to affect brain development, particularly with the advancement of imaging technologies such as MRI. Despite growing interest, the literature remains fragmented and lacks systematic synthesis.

**Methods:**

We performed a bibliometric analysis of 6,446 English-language articles and reviews in the Web of Science Core Collection (1984–2025), using VOSviewer, CiteSpace, SCImago Graphica, and Excel to map knowledge structures, research hotspots, and collaborative networks. A PubMed dataset (*n* = 6,389; 1984–2025) provided validation, comparing annual publication trends and high-frequency keyword structures.

**Results:**

The annual publication trends showed strong concordance between the WoSCC and PubMed datasets (Pearson r = 0.988, R^2^ = 0.976). Keyword co-occurrence analysis identified four primary clusters: “Technology & Development”, “Function & Regulation”, “Risk & Pathology”, and “Hemodynamics & Perfusion”. In the WoSCC keyword co-occurrence network, representative high-TLS keywords included “MRI” (TLS = 2799), “functional connectivity” (TLS = 1,224), “dementia” (TLS = 1,425), and “hemodynamics” (TLS = 1,036). The United States, Germany, and the United Kingdom were the leading contributors, with prominent institutions including the University of Toronto and Harvard Medical School. Citation bursts and recent keywords such as “artificial intelligence” reflect the technological evolution of the field.

**Conclusion:**

This study provides a systematic overview of HBA research trends and thematic evolution. The sustained growth in publications reflects increasing academic attention. Findings offer insights for researchers, clinicians, and policymakers, emphasizing future directions including AI integration and multi-organ network modeling.

## 1 Introduction

The heart–brain axis (HBA) represents the bidirectional interactions between cardiovascular and central nervous systems, which are critical for understanding neurological and cardiovascular comorbidities. Cardiovascular diseases such as arrhythmia and heart failure often accompany cognitive or emotional disturbances, and conversely, neurological disorders can impact cardiac function (1–3). This holistic perspective has prompted a shift from organ-independent models to systems medicine frameworks (4).

Children with congenital heart disease (CHD) may exhibit brain developmental abnormalities, including restricted fetal brain growth and preoperative white matter damage, which can contribute to postoperative neurodevelopmental delays (5–10). The cardiocerebrovascular axis has been increasingly studied across diverse disorders, from stroke, Alzheimer's disease, and heart failure (11) to pediatric neurodevelopmental disorders, anxiety, depression, and autism spectrum disorder (12–14), highlighting the need for advanced assessment tools and imaging technologies (15, 16).

Magnetic resonance imaging (MRI) has become a core tool for assessing brain structural and functional changes in HBA research, particularly in pediatric and adolescent populations. Techniques including structural MRI, functional MRI, diffusion tensor imaging, tensor-based morphometry, and functional connectivity mapping have been used to analyze brain developmental trajectories, preoperative structural abnormalities, and postoperative neurodevelopmental outcomes (9, 17–24).

Recent bibliometric studies in neurology and pediatric cardiology have demonstrated the value of this approach for mapping research landscapes and identifying emerging hotspots, while disease-burden-oriented bibliometric work in cancer suggests that publication patterns may partly correspond to clinical burden (25–27). Despite the rapid growth in MRI-based studies on cardiocerebrovascular interactions, systematic bibliometric evidence on this field remains limited. Therefore, this study aimed to construct an MRI-centered bibliometric map of heart–brain axis research to clarify its publication trends, collaboration networks, thematic clusters, intellectual base, and emerging frontiers.

## 2 Materials and methods

The primary dataset for this bibliometric analysis was retrieved from the Web of Science Core Collection (WoSCC) and covered the period from 1984 to 22 September 2025. The search strategy was designed to capture cross-disciplinary research on congenital heart disease, neurodevelopmental or cognitive outcomes, and MRI-based brain imaging. To ensure that the included literature matched the technical and conceptual scope of this study, we used Boolean logic to exclude two major categories of records: (1) studies whose main contribution lay in MRI technical methodology *per se* rather than in neuroscience or clinical applications; and (2) studies focusing on primary brain-only neurological diseases without a clear heart–brain axis component. This strategy was intended to optimize thematic relevance and better reveal the internal knowledge structure of heart–brain axis research.

The dataset was deliberately constructed as an MRI-centered literature set rather than an all-modality heart–brain axis dataset. The search strategy combined cardiovascular terms, brain/neurodevelopmental or cognitive terms, and MRI-related imaging terms to ensure a coherent technical and conceptual basis. CT/CT perfusion, EEG, PET-only studies, vascular ultrasound/TCD studies, and general multimodal cardiovascular imaging studies were not used as primary search targets because they differ from MRI-based neuroimaging in measurement principles, clinical indications, and bibliometric communities. Including all modalities in the same primary dataset would have broadened the scope and reduced the interpretability of MRI-specific keyword clusters and collaboration networks.

**Figure 1:**
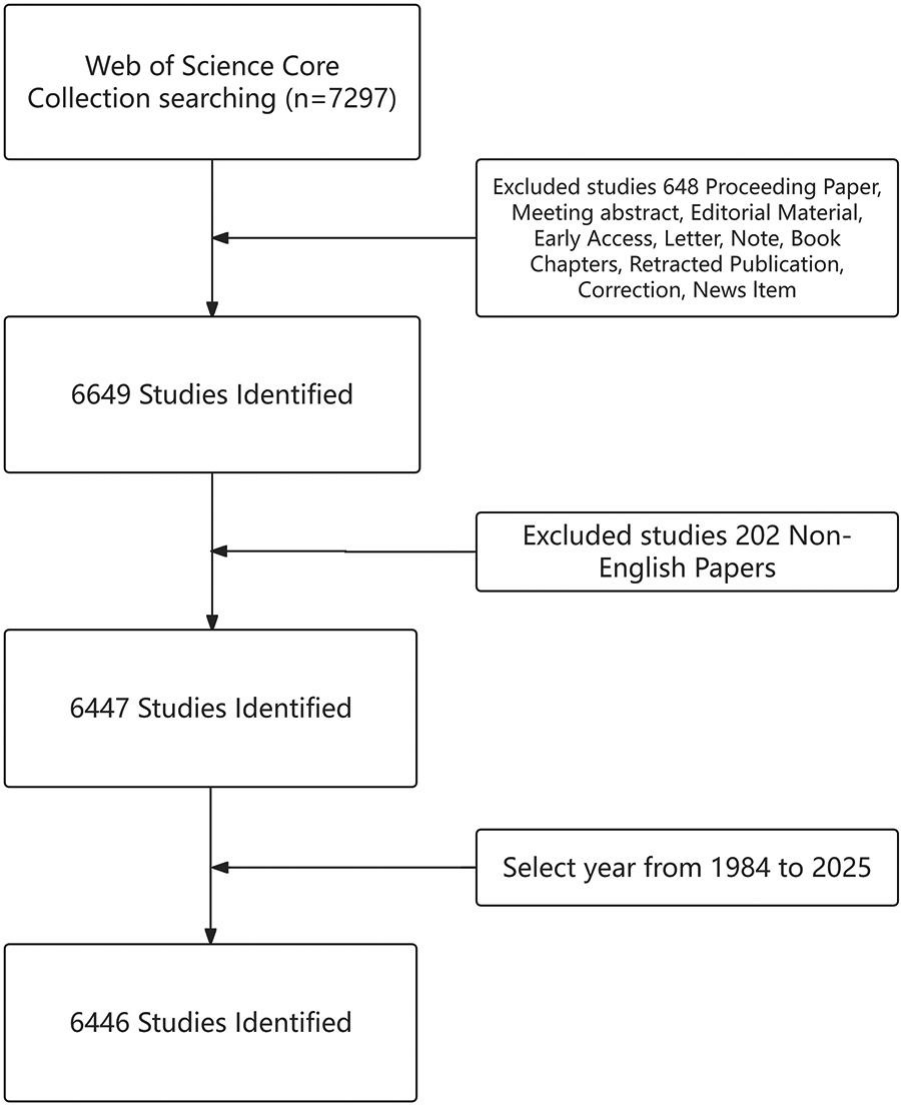
Flowchart of the literature search and study-selection process.

In addition, this bibliometric study adopted a multi-database design. A parallel search was conducted in PubMed using an adapted version of the same query, limited to journal articles and reviews in English from 1984 to 22 September 2025. PubMed records were used to validate the robustness of the WoSCC dataset by comparing annual publication counts and high-frequency keywords/MeSH terms. The complete search strategies for both databases are provided in the Supplementary Material, and the detailed operational workflow is illustrated in Figure 1.

This article primarily utilized VOSviewer (version 1.6.20), CiteSpace (version 6.4.R1), SCImago Graphica (version 1.0.51.0), and Microsoft Excel (Version 2508) for data processing and graph construction. VOSviewer was used to construct co-occurrence, co-authorship, citation, and co-citation networks. CiteSpace was employed to identify highly prominent keywords and track the evolution of research frontiers. SCImago Graphica was used to create a country co-authorship overlay map incorporating a world map. Excel was primarily utilized for organizing, filtering, and integrating charts and graphs of literature data. In VOSviewer maps, node size represents item frequency or weight, link thickness reflects association strength, and color indicates cluster membership or average publication year depending on the map type; in CiteSpace burst maps, burst strength and red bars indicate the intensity and active period of sudden increases in attention.

In the main keyword co-occurrence analysis, the unit of analysis was set as all keywords exported from WoSCC, including both author keywords and index keywords. A minimum frequency threshold of 50 was set to construct network graphs, overlay graphs, and density graphs, aiming to identify high-frequency research topics and conceptual clusters within the field. Because different keyword sources may provide complementary but not identical representations of research topics, we additionally performed an author keywords co-occurrence analysis as a supplementary keyword-source validation. In this supplementary analysis, a minimum occurrence threshold of 20 was applied; among 12,218 author keywords, 146 met the threshold and were included in the network. In author co-authorship analysis (co-authorship: authors), a minimum publication threshold of 5 articles was set, resulting in the identification of 495 authors who met the threshold.

At the institutional level (co-authorship: organizations), a minimum publication threshold of 10 articles was set, with 350 institutions meeting the criteria. At the national level (co-authorship: countries), a minimum of 5 publications per country was set, ultimately including 65 countries and regions. The citation analysis encompassed three dimensions: documents, sources, and authors, with respective minimum thresholds for citations or publication quantity set, identifying high-impact documents and authors. The co-citation analysis covered cited references, cited sources, and cited authors, with minimum citation frequencies set at 20, 50, and 50 respectively, to construct a core literature and theoretical foundation map. Additionally, based on CiteSpace, the Top 30 emerging keywords from 1984 to 2025 were identified, revealing the dynamic evolution trend of research hotspots. The term “average publication time” mentioned in this article refers to the weighted average of all publication years for each country/institution/author (or keyword). For example, “2012.5” indicates close to the middle of 2012 (approximately June). Detailed VOSviewer and CiteSpace parameters, including units of analysis, thresholds, normalization and counting methods, clustering settings, keyword standardization procedures, and burst analysis settings, are summarized in Supplementary Table S1. Thematic labels of clusters were assigned based on high-frequency keywords, TLS values, and representative records within each cluster, and were subsequently reviewed by another author; disagreements were resolved through discussion and consensus.

Citation and co-citation analyses were based on citation information exported from WoSCC without manual author-level citation selection. Systematic self-citation correction was not applied, as the purpose was to characterize the naturally indexed citation structure of the field rather than to rank individual authors or publications after self-citation adjustment.

## 3 Results

### 3.1 Annual publications

**Figure 2:**
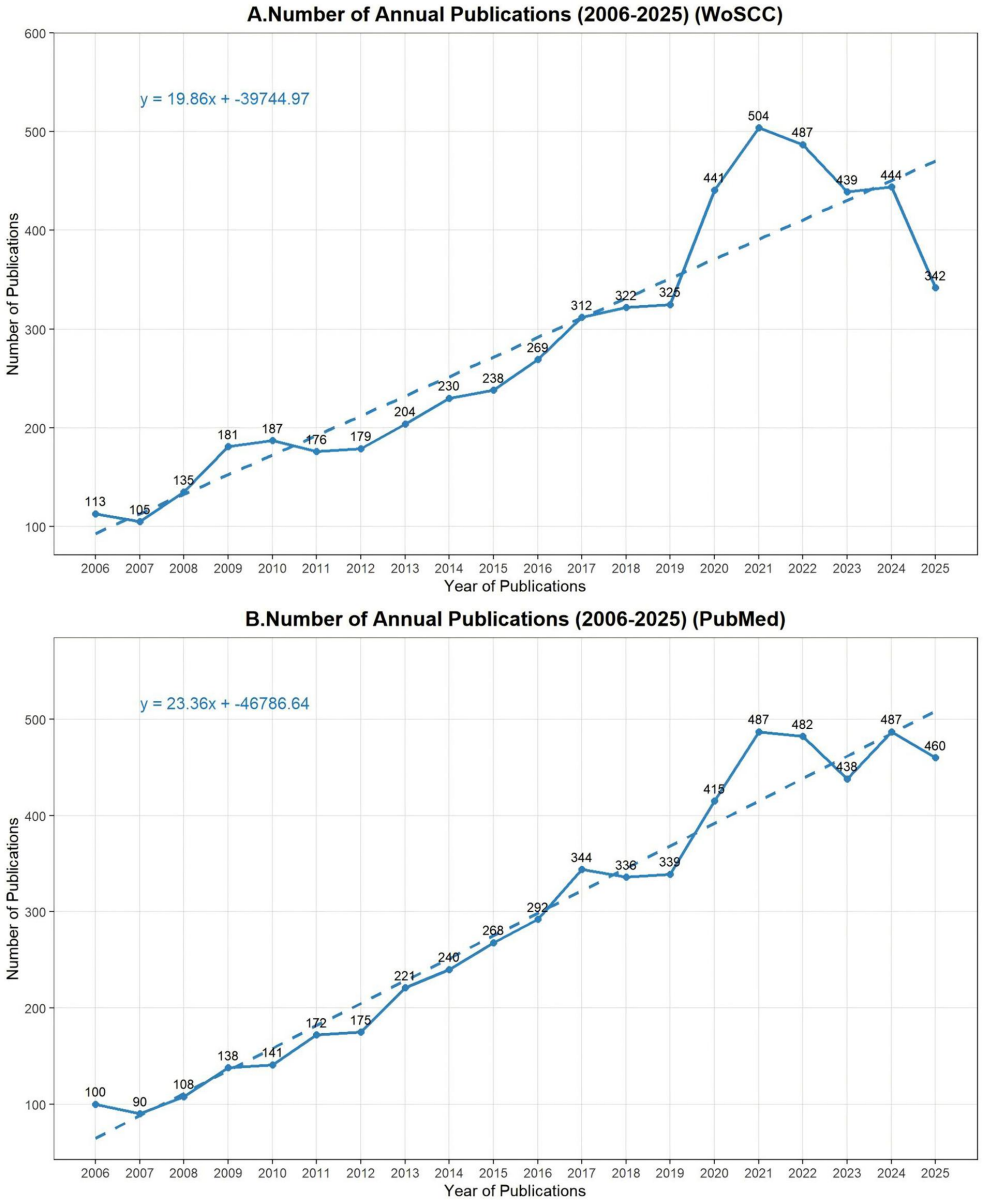
Annual publication trends from 2006 to 2025 in **(A)** WoSCC and **(B)** PubMed.

Over the past 42 years (1984–2025), 6,446 articles were retrieved from the Web of Science Core Collection (WoSCC), with an average annual output of approximately 153.5 publications. As part of the multi-database validation, using an adapted version of the same search strategy, PubMed yielded 6,389 journal articles and reviews over the identical period. The annual publication counts from WoSCC and PubMed showed an excellent temporal concordance (Pearson r = 0.988, R^2^ = 0.976, t = 40.04, *p* = 6.78 × 10⁻3⁴). Minor year-to-year differences, with WoSCC being higher in some years and PubMed in others, are likely attributable to differences in database coverage, indexing policies, and document-type definitions. For both databases, the apparent decline in 2025 reflects the fact that data collection was truncated on 22 September 2025, before all publications for that year had been fully indexed. Within the past 20 years (2006–2025), 2007 (*n* = 105) had the lowest annual output, whereas 2021 (*n* = 504) had the highest. Overall, WoSCC annual publications exhibited a steady growth trend, with a linear regression equation of Y = 19.86X−39,744.97. The detailed trends for WoSCC and PubMed are presented in Figure 2.

### 3.2 Co-occurrence of all keywords

Using a minimum occurrence threshold of ≥50, 189 high-frequency keywords were identified in the WoSCC dataset. Applying the same threshold to PubMed yielded 158 author/all keywords and 136 MeSH terms; among these, 108 (68.1%) and 92 (67.6%), respectively, were identical to or close synonyms of the WoSCC terms. This substantial overlap indicates that the major thematic structure captured in WoSCC is largely reproducible in an independent biomedical database.

After analyzing all keywords, the literature was categorized into four main research directions. Cluster 1 includes high-frequency keywords such as congenital heart disease, brain development, MRI, preterm infants, neurodevelopment, “deep learning,” “artificial intelligence,” and “segmentation,” primarily focusing on “MRI-driven ischemic brain disease and brain structural damage,” “brain development in children with congenital heart disease” and “artificial intelligence and image analysis”, which can be broadly summarized as “Technology & Development”. Cluster 2 includes high-frequency keywords such as heart rate variability, amygdala, insula, functional connectivity, autonomic nervous system, and emotion regulation, mainly involving “cardiovascular-cerebral functional connectivity mechanisms and the value of heart rate variability (HRV) indicators” and “functional networks and key brain regions in emotional regulation mechanisms”, corresponding to the “Function & Regulation”. Cluster 3 includes high-frequency keywords such as dementia, Alzheimer's disease, hypertension, white matter hyperintensities, and cognitive impairment, which frequently appear in this cluster, reflecting the impact mechanism of cardiovascular factors on cognitive function in old age and the association between cerebral small vessel disease and white matter lesions, as “Risk & Pathology”. Cluster 4 includes high-frequency keywords such as cerebral blood flow, hemodynamics, transcranial Doppler, phase-contrast MRI, cerebrovascular reactivity, and wall shear stress, primarily involving “cerebral blood flow dynamics” and “the application of multimodal imaging in blood flow assessment”, representing the “Hemodynamics & Perfusion”. The supplementary author keywords co-occurrence analysis included 146 keywords with at least 20 occurrences and showed substantial thematic overlap with the main all-keyword network, including MRI/neuroimaging methods, functional connectivity and autonomic regulation, vascular-cognitive pathology, and hemodynamic/perfusion-related topics (Supplementary Figure). Representative high-TLS keywords for each major cluster, derived from the WoSCC keyword co-occurrence network, are summarized in (Supplementary Table S2).

**Figure 3:**
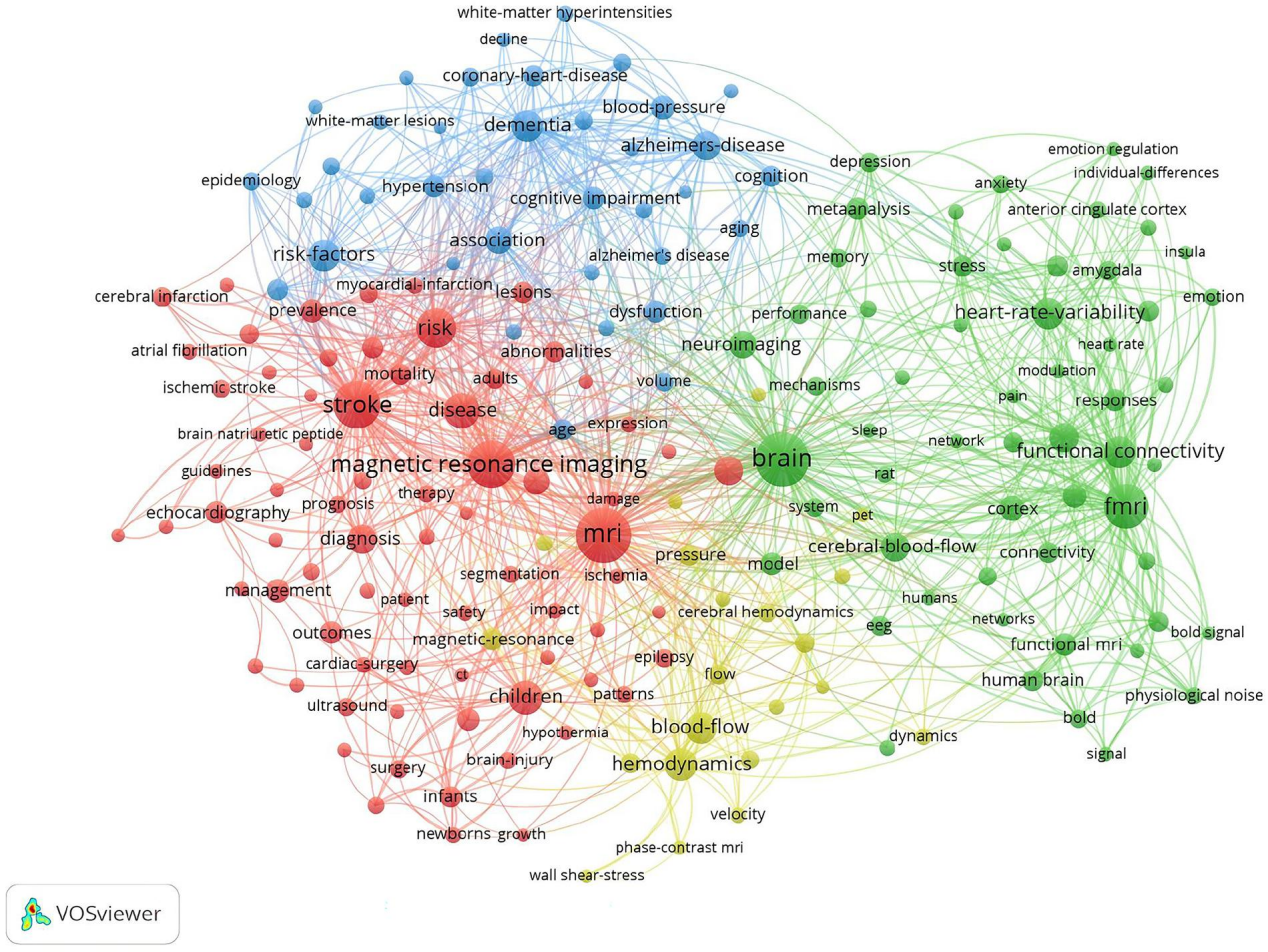
Keyword co-occurrence network of MRI-related heart–brain axis research. Node size represents keyword occurrence, link thickness represents co-occurrence strength, and colors indicate algorithm-derived clusters: red, Cluster 1; green, Cluster 2; blue, Cluster 3; and yellow, Cluster 4.

**Figure 4:**
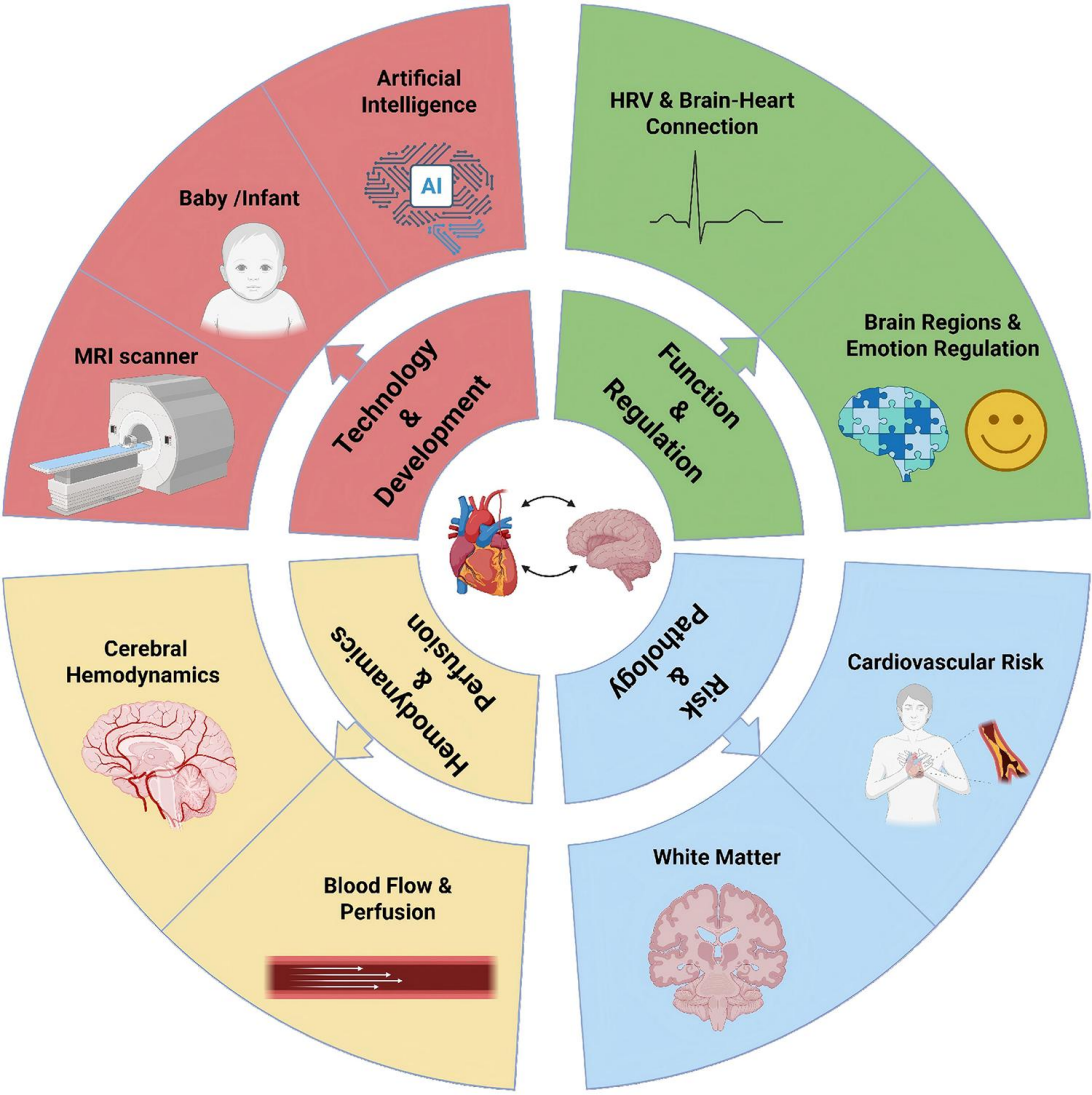
Schematic overview of the four major thematic clusters in MRI-related heart–brain axis research. Created in BioRender. Guo, Y. (2026) https://BioRender.com/49leui2

In the keyword network map, larger nodes and thicker links indicate more frequent keywords and stronger co-occurrence relationships, while colors represent algorithm-derived clusters. These visualization patterns supported the classification of MRI-related HBA research into four major thematic directions. An overview of Clusters 1–4 is shown in (Figures 3, 4).

### 3.3 Co-author with countries/regions and organizations

**Figure 5:**
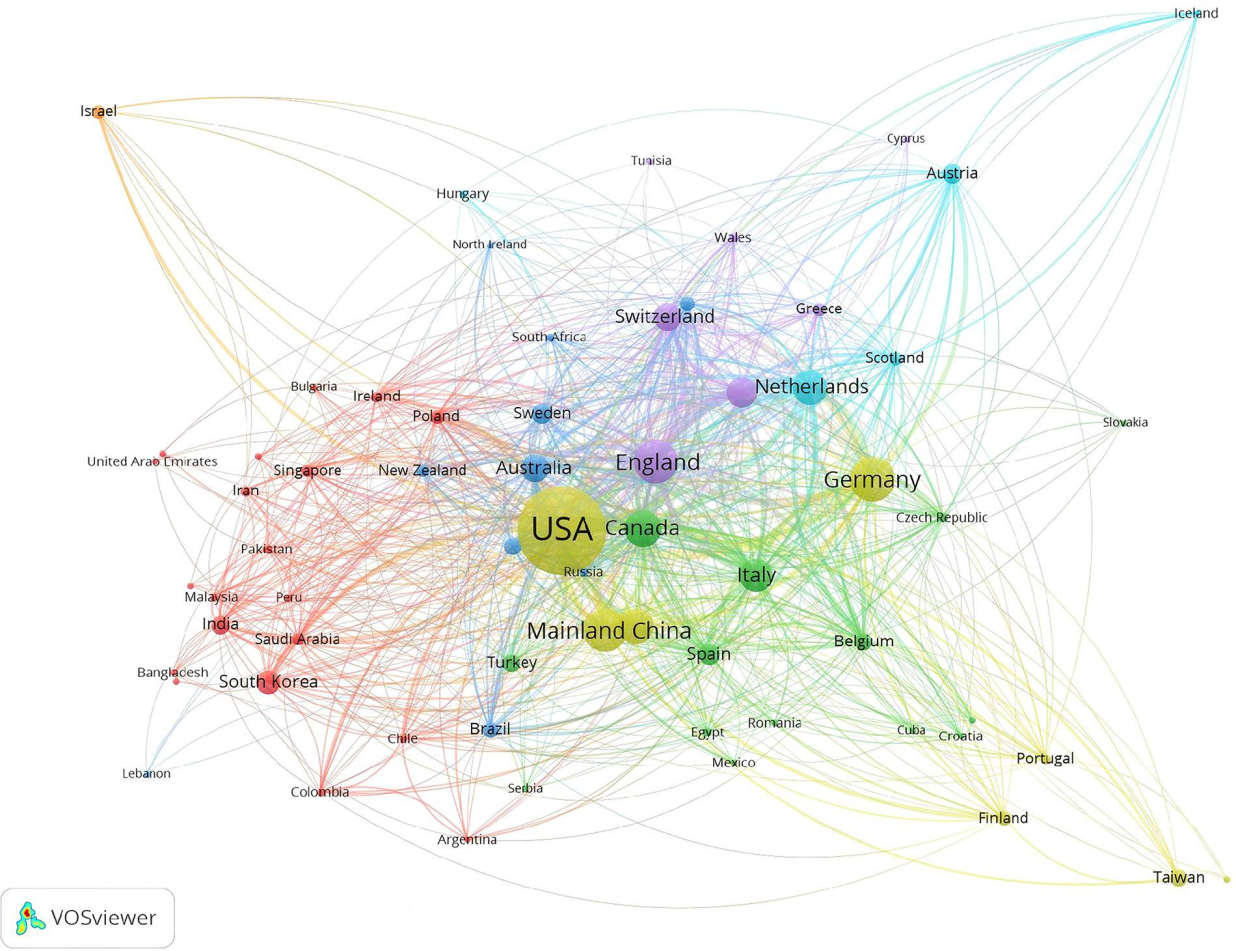
Country/region co-authorship network. Node size represents publication output, and link thickness represents collaboration strength.

**Figure 6:**
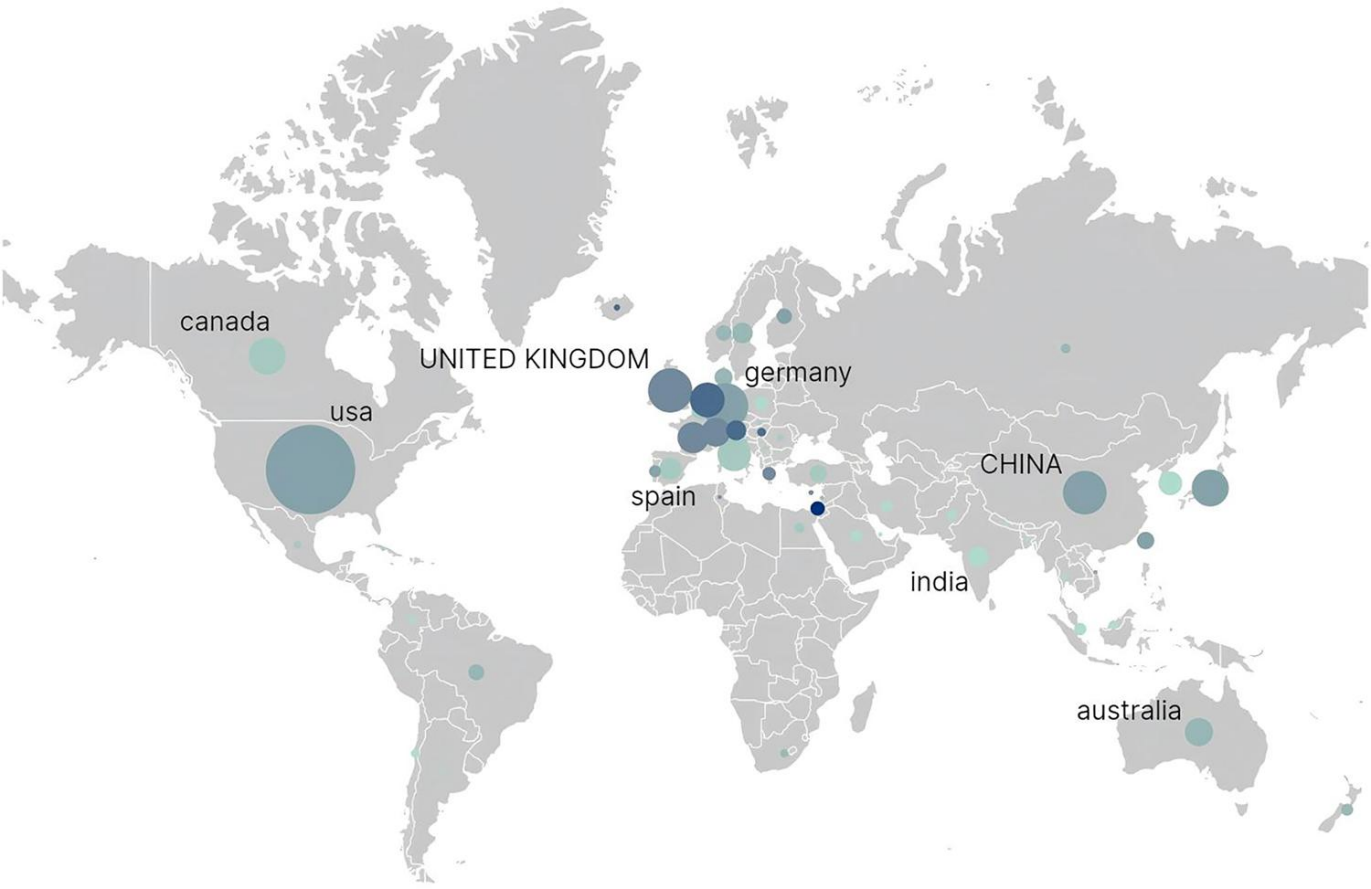
Geographic distribution of country/region co-authorship generated using SCImago Graphica. Circle size represents publication output.

In the analysis of cooperation at the national and regional level, a total of 65 countries and regions met the minimum publication criteria (≥5 articles). The data reveal that the United States stands out in terms of research output and influence, with a total of 2,442 published articles, 123,844 cumulative citations, and a Total Link Strength (TLS) of 1,481. Germany (646 articles, 34,704 citations) and the United Kingdom (597 articles, 30,219 citations) are prominent in the European region. China ranks fourth with 583 articles and a TLS of 291. Canada, with 425 published articles and 18,252 citations, occupies the fifth position, with a cooperation network TLS of 548 (Figures 5, 6; Table 1).

**Table 1:** Top 10 countries/regions, organizations, and authors in the co-authorship analyses.

Rank	Country/Region	Documents	Citations	TLS	Links	Year	ACPP
1	USA	2,442	123,844	1,481	63	2015.3	50.71
2	Germany	646	34,704	720	50	2015.6	53.72
3	England	597	30,219	833	59	2016.4	50.62
4	China	583	7,931	291	41	2020.3	13.60
5	Canada	425	18,252	548	55	2016.5	42.95
6	Japan	417	10,994	166	38	2014.5	26.36
7	Netherlands	366	17,644	496	47	2015.9	48.21
8	Italy	345	13,713	455	51	2016.9	39.75
9	France	296	10,394	364	48	2015.7	35.11
10	Switzerland	253	16,238	424	47	2015.9	64.18
Rank	Organization	Documents	Citations	TLS	Links	Year	ACPP
1	University of Toronto	142	4,188	367	137	2017.9	29.49
2	Harvard Medical School	139	3,948	401	136	2020.8	28.40
3	Boston University	122	5,886	383	87	2017.1	48.25
4	Harvard University	111	8,518	231	98	2011.2	76.74
5	Massachusetts General Hospital	102	8,474	242	97	2014.8	83.08
6	University of Pennsylvania	93	5,263	227	99	2016.7	56.59
7	University of California, Los Angeles	91	5,822	127	71	2013.7	63.98
8	Columbia University	90	4,979	228	106	2015.9	55.32
9	University of California, Davis	89	4,880	287	79	2016.8	54.83
10	King's College London	87	2,359	182	107	2019.8	27.11
Rank	Author	Documents	Citations	TLS	Links	Year	ACPP
1	Seshadri S.	65	5,687	353	47	2018.9	87.49
2	DeCarli C.	58	5,414	304	52	2017.8	93.34
3	Beiser A. S.	36	1,406	212	35	2019.3	39.06
4	Au R.	27	1,379	146	30	2017.3	51.07
5	Himali J. J.	27	1,522	170	38	2018.7	56.37
6	Thayer J. F.	25	3,097	52	12	2017.0	123.88
7	Biessels G. J.	24	686	75	20	2021.1	28.58
8	Seed M.	24	910	85	16	2020.0	37.92
9	Vasan R. S.	24	1,381	138	26	2018.8	57.54
10	Kumar R.	23	725	74	9	2016.3	31.52

TLS, total link strength, ACPP, average citations per publication.

**Figure 7:**
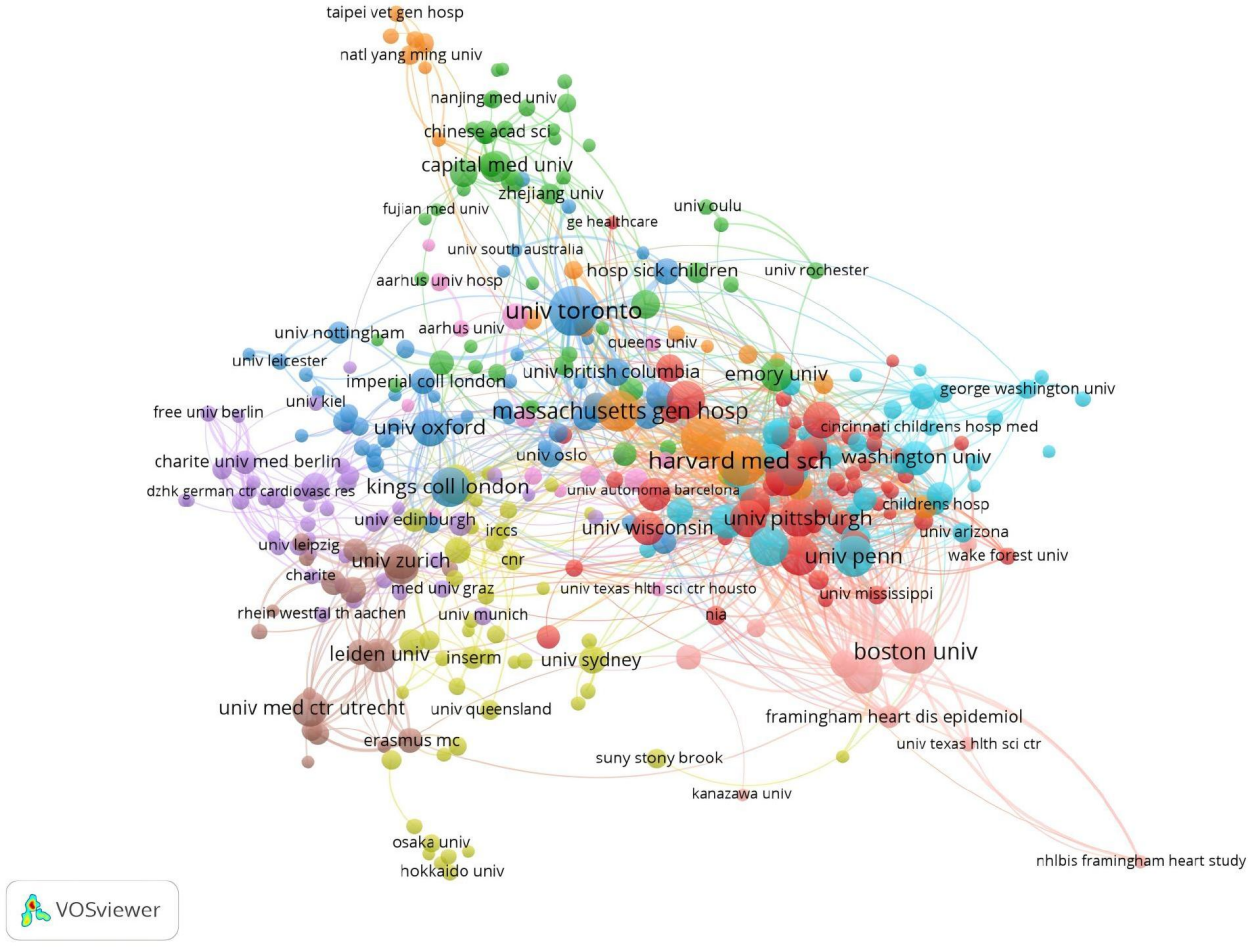
Organization co-authorship network. Node size represents publication output, and link thickness represents collaboration strength between organizations.

In the analysis of cooperation at the organizational level, a total of 350 organizations met the minimum publication criteria (≥10 articles). The University of Toronto ranks first with 142 articles, 137 links, and a total connection strength of 367. Harvard Medical School closely follows, with 139 articles and the highest total connection strength (401). Boston University also ranked prominently, publishing 122 articles with a total connection strength of 383 and a citation count of 5,886. In addition, Harvard University and Massachusetts General Hospital also ranked in the top five with 111 and 102 articles, respectively, with a total citation count of over 8,400 (Figure 7).

### 3.4 Co-author with authors

**Figure 8:**
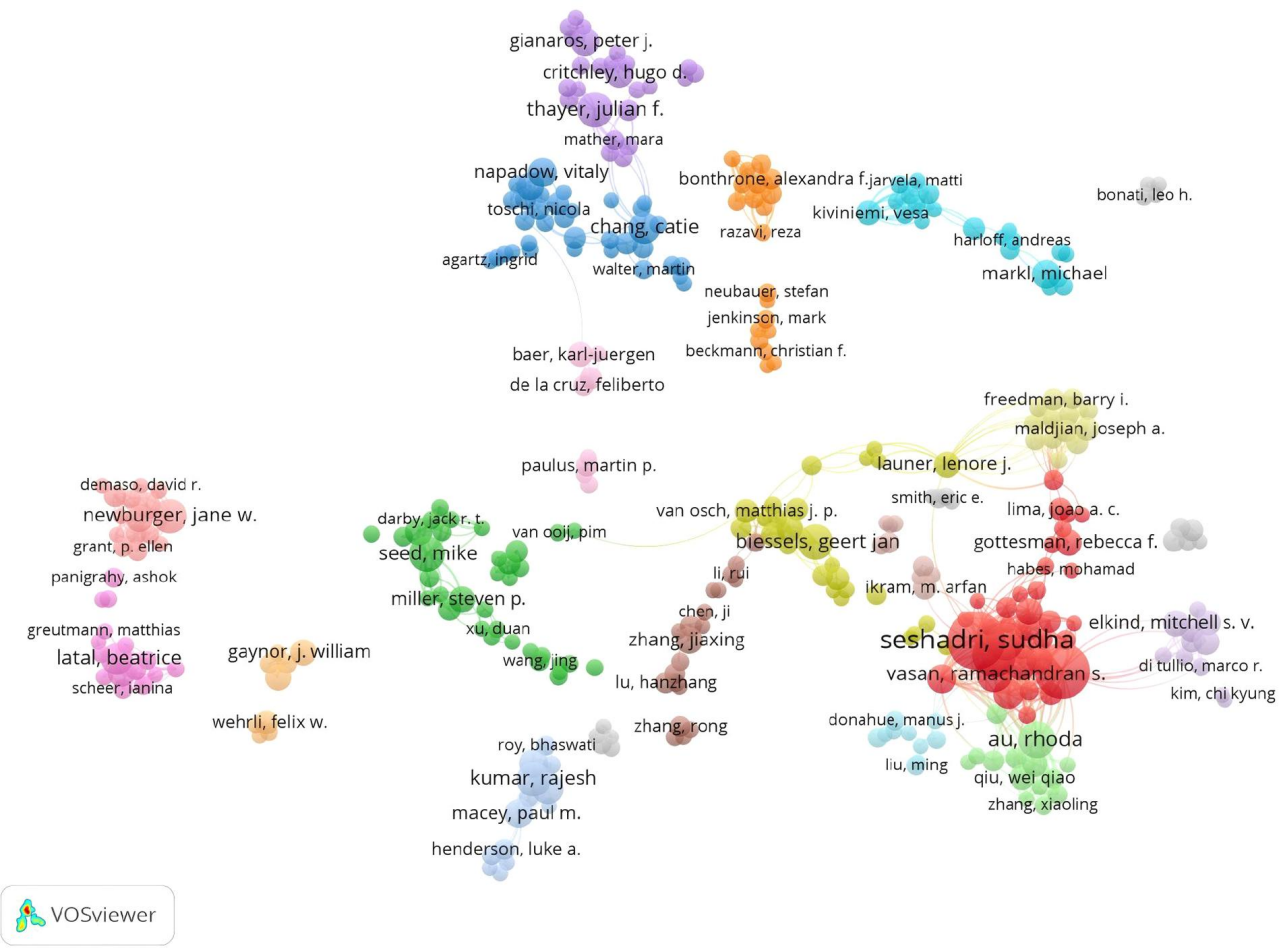
Author co-authorship network. Node size represents the number of publications by each author, and link thickness represents collaboration strength between authors.

At the author collaboration level, a total of 495 authors have published no fewer than 5 articles on the research topic over the past two decades. According to the co-authorship network, Sudha Seshadri has published 65 articles with a total of 5,687 citations, boasting the highest overall connectivity strength (353). Charles DeCarli follows closely behind, with 58 articles and 5,414 citations, achieving a total connectivity strength of 304, also making him a high-impact core author. Alexa Beiser ranks third with 36 articles and a total connectivity strength of 212, followed by Rhoda Au and Jayandra J. Himali, both of whom have published 27 articles, with total connectivity strengths of 146 and 170 (Figure 8).

### 3.5 Co-citation cited sources and references

**Figure 9:**
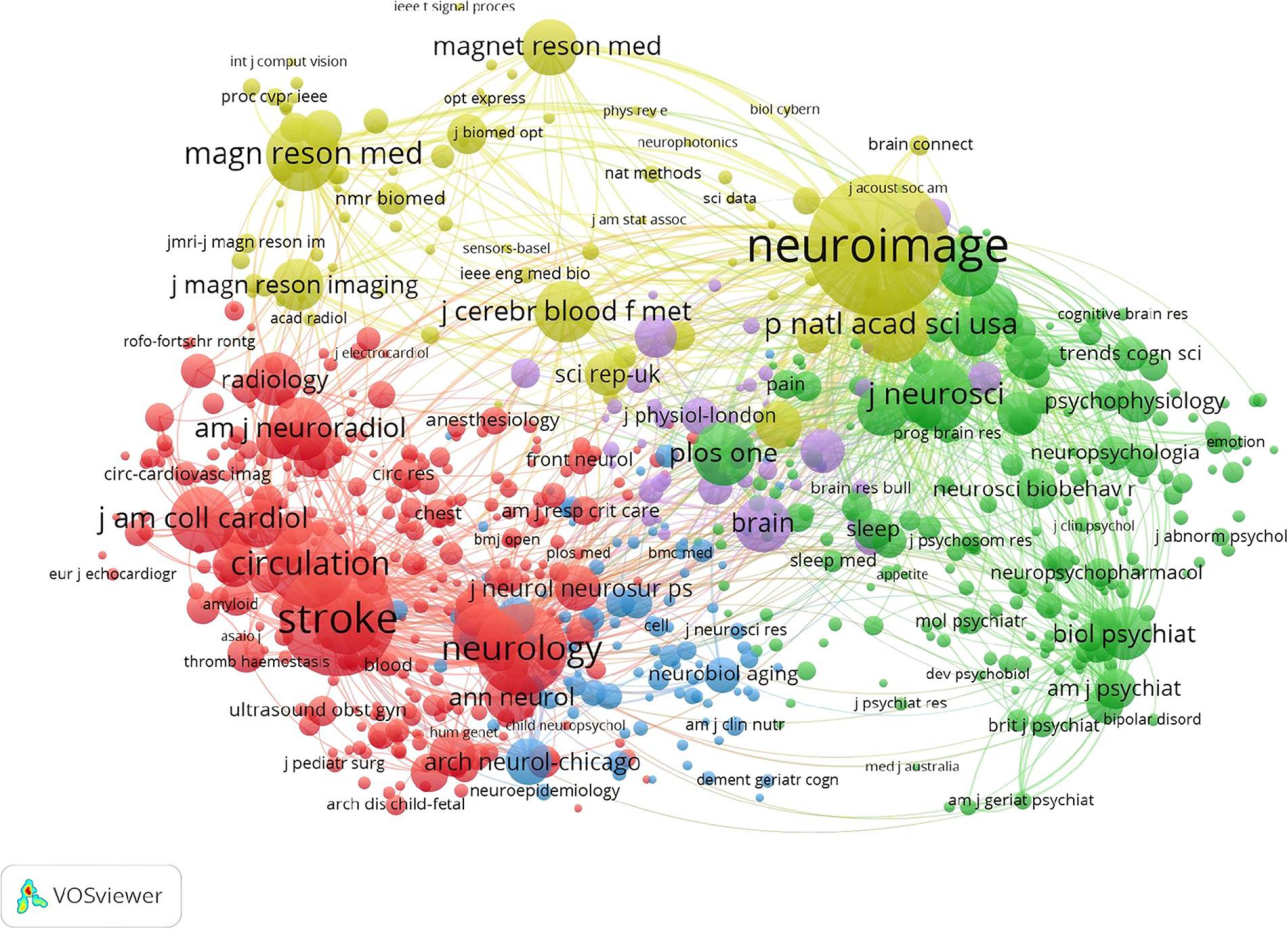
Co-citation network of cited sources. Node size represents the co-citation frequency of each cited source, link thickness represents co-citation strength between sources, and colors indicate co-citation clusters.

In the analysis of co-citation of cited sources, a total of 755 sources met the threshold of “citation count ≥50”. Co-citation maps were interpreted as indicators of the intellectual base of the field rather than simple publication productivity. Among these frequently co-cited sources, the top five journals were “NeuroImage”, “Stroke”, “Neurology”, “Circulation”, and “Proceedings of the National Academy of Sciences of the United States of America (PNAS)”, all of which exhibit extremely high co-citation frequencies and total link strengths. Among them, “NeuroImage” ranked first with 13,343 co-citations and a total link strength of 769,654, followed closely by “Stroke” with 8,975 co-citations and a total link strength of 399,090. “Neurology” had 5,706 co-citations and a link strength of 324,304. “Circulation” had 4,918 co-citations and a total link strength of 238,027. “PNAS” had 3,683 co-citations and a link strength of 268,909 (Figure 9; Table 2).

**Table 2:** Top 10 cited sources by co-citation frequency.

Rank	Source	Citations	Total link strength	Links
1	NeuroImage	13,343	769,654	753
2	Stroke	8,975	399,090	753
3	Neurology	5,706	324,304	754
4	Circulation	4,918	238,027	754
5	PNAS	3,683	268,909	753
6	Magnetic Resonance in Medicine	3,551	160,159	730
7	Journal of Neuroscience	3,447	265,802	747
8	The New England Journal of Medicine	2,951	149,751	753
9	Journal of the American College of Cardiology	2,935	149,716	749
10	American Journal of Neuroradiology	2,758	114,322	746

This table shows the specific situations of the top 10 co-citation cited sources (journals).

**Figure 10:**
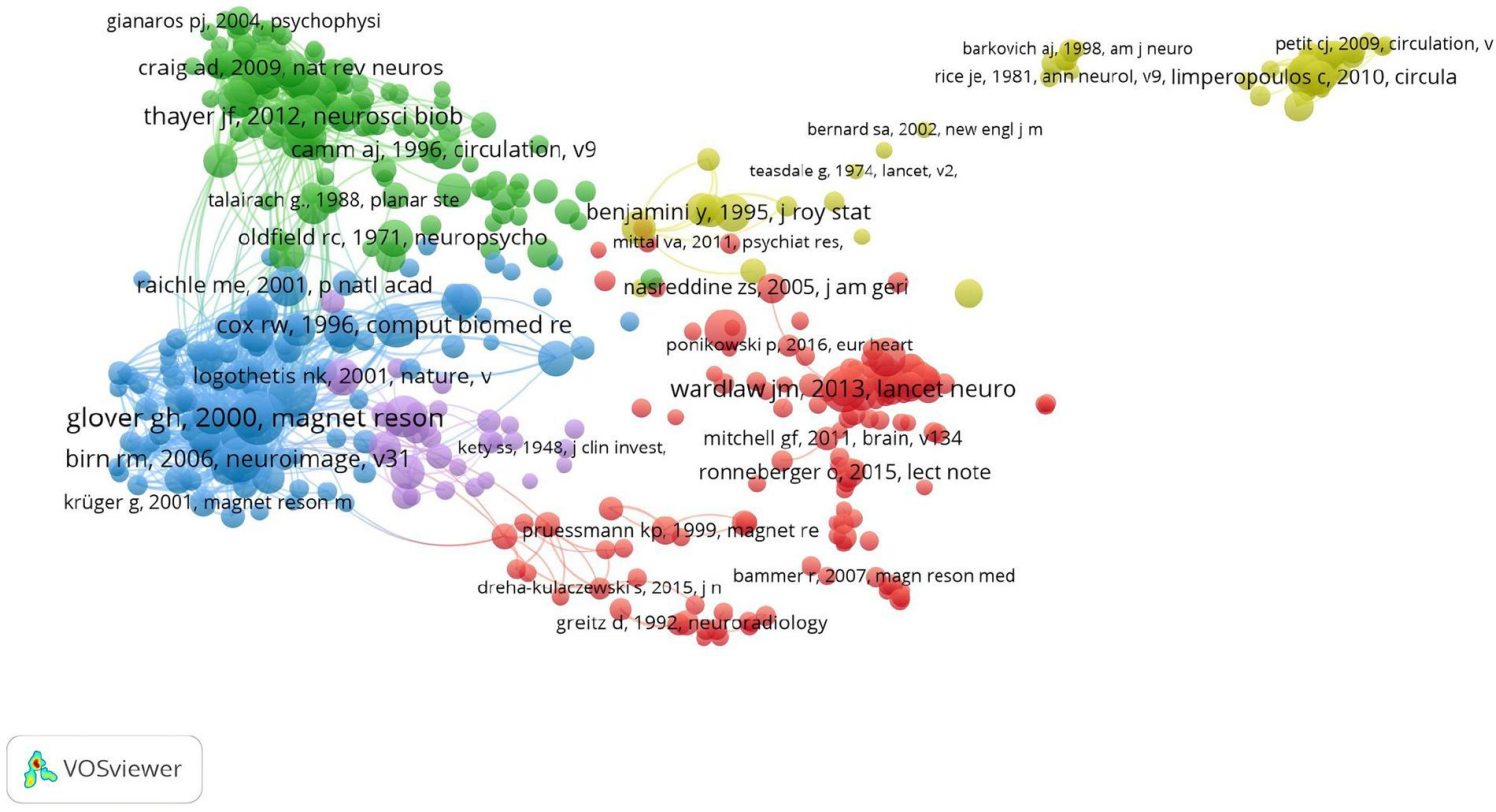
Co-citation network of cited references. Node size represents the co-citation frequency of each cited reference, link thickness represents co-citation strength between references, and colors indicate co-citation clusters.

In the analysis of co-citation of cited references, a total of 473 articles met the threshold of at least 20 co-citations. The paper with the highest co-citation frequency was the one published by Glover et al. in 2000 in Magnetic Resonance in Medicine (co-cited 182 times). The second highest is the paper by Biswal et al. published in 1995 (co-cited 149 times). The third highest is the review article by Wardlaw et al. published in 2013 in Lancet Neurology (co-cited 138 times) (Figure 10; Table 3).

**Table 3:** Top 10 cited references by co-citation frequency.

Rank	First author	Publication year	Title	Journal	Total citations
1	Glover G.H.	2000	Image-based method for retrospective correction of physiological motion effects in fMRI: RETROICOR	Magnetic Resonance in Medicine	182
2	Biswal B.	1995	Functional connectivity in the motor cortex of resting human brain using echo-planar mri	Magnetic Resonance in Medicine	149
3	Wardlaw J.M.	2013	Neuroimaging standards for research into small vessel disease and its contribution to ageing and neurodegeneration	The Lancet Neurology	138
4	Thayer J.F.	2012	A meta-analysis of heart rate variability and neuroimaging studies: Implications for heart rate variability as a marker of stress and health	Neuroscience & Biobehavioral Reviews	132
5	Cox R.W.	1996	AFNI: Software for Analysis and Visualization of Functional Magnetic Resonance Neuroimages	Computers and Biomedical Research	130
6	Birn R.M.	2006	Separating respiratory-variation-related fluctuations from neuronal-activity-related fluctuations in fMRI	NeuroImage	129
7	Chang C.	2009	Influence of heart rate on the BOLD signal: The cardiac response function	NeuroImage	120
8	Ogawa S.	1990	Brain magnetic resonance imaging with contrast dependent on blood oxygenation.	Proceedings of the National Academy of Sciences	117
9	Folstein M.F.	1975	"Mini-mental state” a practical method for grading the cognitive state of patients for the clinician	Journal of Psychiatric Research	113
10	Beissner F.	2013	The Autonomic Brain: An Activation Likelihood Estimation Meta-Analysis for Central Processing of Autonomic Function	Journal of Neuroscience	109

This table shows the specific situations of the top 10 co-citation cited references.

### 3.6 Burst keywords and references

**Figure 11:**
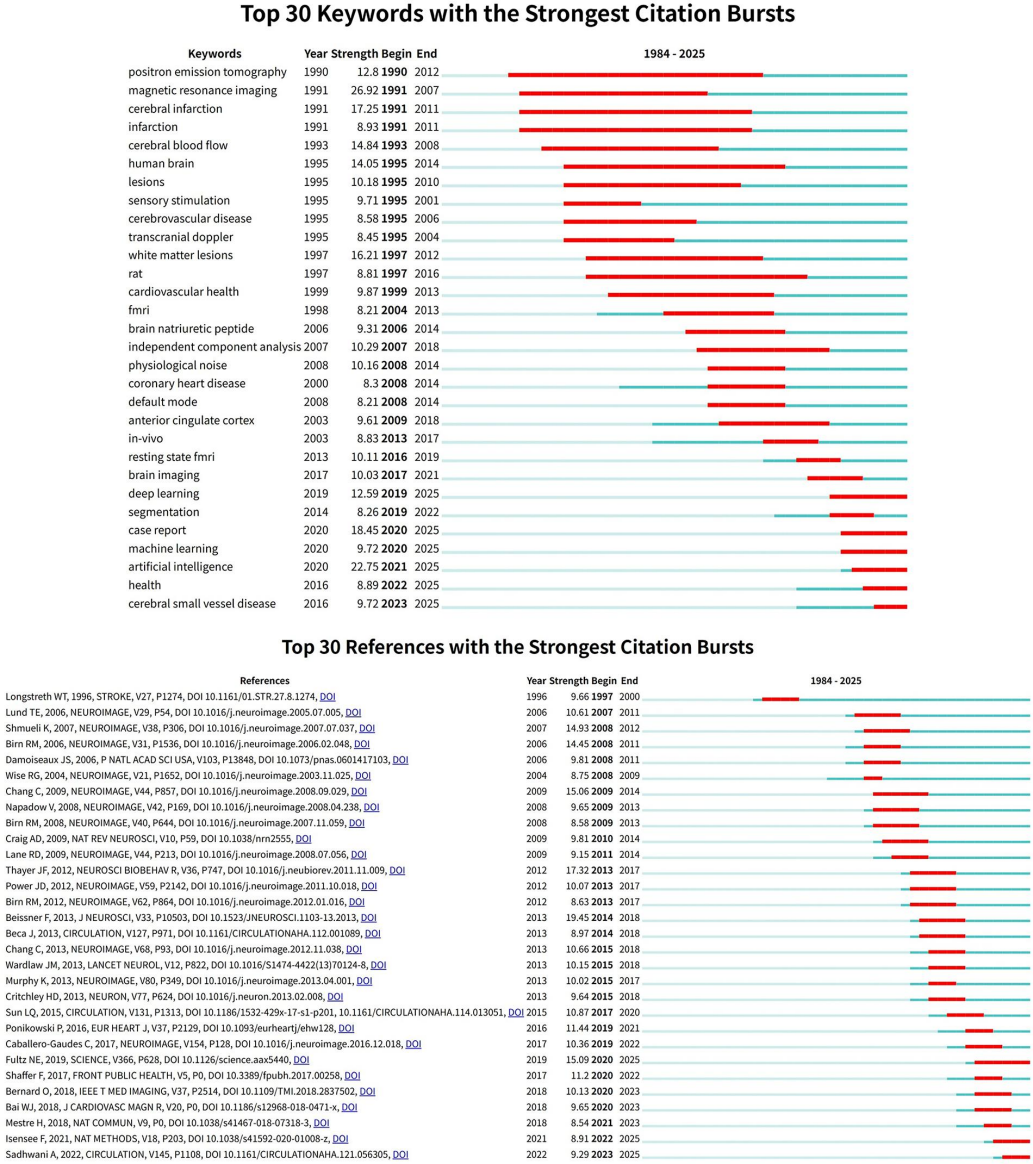
Top 30 keywords and references with the strongest citation bursts generated by CiteSpace. “Year” indicates the year associated with each keyword or reference, “Strength” represents the relative burst intensity calculated by CiteSpace, and “Begin” and “End” indicate the starting and ending years of the burst period. The red bars on the timeline indicate the active burst period between 1984 and 2025.

We used CiteSpace to generate the images of top 30 keywords and references with the strongest citation bursts. They showed a sudden and sharp increase in academic attention to keywords and specific literature during a specific period. “Year” represents the average year in which the keyword or reference appears in the set, while “Strength” is the relative strength calculated by CiteSpace. “Begin” and “End” represent the starting and ending times of the keyword or reference set's appearance period. The red part of the timeline on the right reflects its concentrated appearance time from 1984 to 2025 (Figure 11). Burst maps were used to identify time-specific research frontiers rather than overall importance rankings.

Early emerging keywords focused on imaging techniques and descriptions of pathological processes of cerebral blood flow, such as “positron emission tomography (1990–2012)” and “magnetic resonance imaging (1991–2007)”. Terms like “cerebral infarction” and “cerebral blood flow” reflected the focus on cerebral hemodynamics and ischemic mechanisms. Additionally, entries such as “white matter lesions” (1997–2012) and “human brain” (1995–2014) showed a long-lasting presence.

After entering the 2010s, burst keywords shifted towards brain functional networks and cognitive regulation, represented by “independent component analysis” (2007–2018), “resting state fMRI” (2016–2019), and “default mode” (2008–2014). In recent years, artificial intelligence-related technologies have emerged strongly, such as “deep learning” (2019–2025), “machine learning” (2020–2025), and “artificial intelligence” (2021–2025), among which “artificial intelligence” has the highest burst strength (22.75).

In the analysis of highly emergent references, such as the series of works (28–30), the intensity of emergence was extremely high from 2008 to 2013, focusing on issues such as low-frequency fluctuations, physiological noise, and interference from signal sources in fMRI; while study (31) was the first to systematically propose the existence of the default mode network in a resting state.

**Figure 12:**
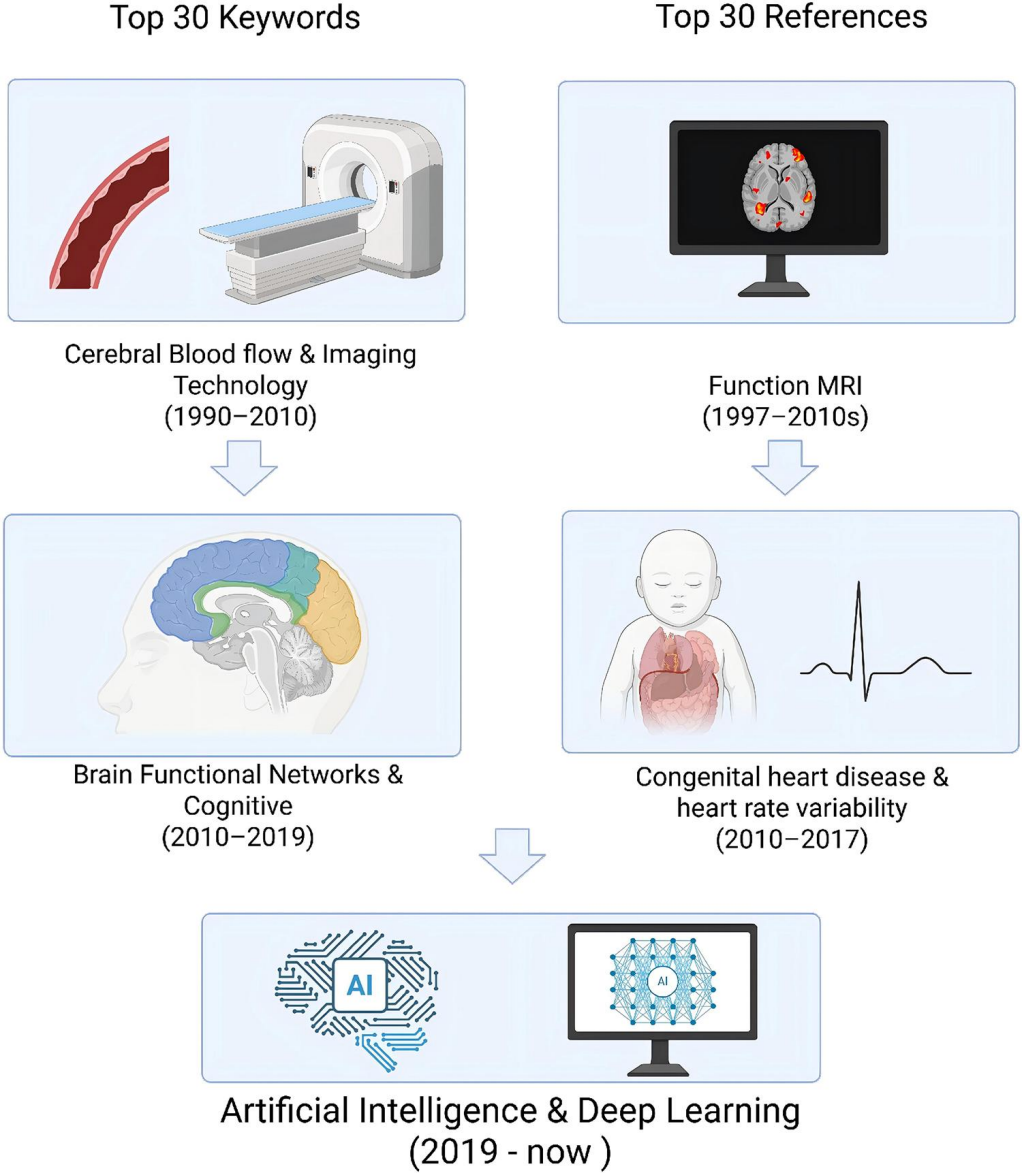
Schematic overview of emerging directions in MRI-related heart–brain axis research, illustrating the evolution of research focus based on the analysis of the top 30 keywords and references with the strongest citation bursts. Created in BioRender. Guo, Y. (2026) https://BioRender.com/gbecuf8

After entering the 2010s, study (32) explored the brain injuries shown in MRI of infants undergoing CHD surgery, while study (33) discussed the predictive value of fetal brain volume for neurodevelopment in children with congenital heart disease. Study (34) explored the association between HRV and fluctuations in resting-state functional connectivity, while study (35) emphasized the importance of HRV. Studies (36, 37) discussed how to eliminate confounding factors in BOLD signals in rest-state fMRI. In recent years, AI-related content has begun to appear in burst literature, with studies (38–40) mentioning the application of deep learning and neural networks in MRI-related research on heart-brain interactions (Figure 12).

## 4 Discussion

This study summarizes MRI-based heart–brain axis research by bibliometric analysis. This line of research has undergone four major developmental phases, with deepening investigations across four key thematic domains: technology, functional assessment, risk evaluation, and hemodynamic characterization. This study provides a systematic review of the research progress within each of these four clusters.

### 4.1 Technology & development (cluster 1)

#### 4.1.1 Ischemic encephalopathy and brain structural damage based on MRI

Structural MRI is the core analytical tool of cluster 1, reflected in the high frequency of appearance of “MRI” and its full name “magnetic resonance imaging”. It is widely used to observe anatomical abnormalities of brain tissue, patterns of disease damage, and developmental differences, and systematically assess changes in gray matter and white matter microstructure, cortical thickness variations, and brain atrophy (41–45). It serves as the technical backbone for imaging research on heart-brain related diseases.

At the disease level, this cluster is highly focused on research related to ischemic brain injury, especially the structural imaging phenotypes of stroke and cerebral infarction (46). The research concentrates on the quantitative description of brain tissue changes in both the acute and chronic phases, emphasizing the value of infarct volume, white matter hyperintensities (WMH) burden, and secondary brain atrophy in assessing disease progression (47). Additionally, recent studies are paying attention to whole brain volume changes, cortical-subcortical structural co-degeneration, and its association with functional recovery or cognitive outcomes (48–50).

It is noteworthy that the research on heart-brain interaction has expanded from the traditional “dual-organ” model to multiple organ axes. Recent studies have shown that cardiac pathology not only directly leads to brain damage but also jointly affects clinical outcomes such as vascular cognitive impairment and dementia with factors such as metabolism (51). From a temporal perspective, this research direction is the earliest formed (from the 1990s to the early 2010s) and has long dominated as a basic structural imaging module in heart-brain axis research.

#### 4.1.2 Brain development in children with CHD

This section exemplifies the core of the “Technology & Development” cluster, applying advanced imaging to a critical clinical population. Cluster 1 is highly focused on the pediatric population, as evidenced by the frequent appearance of the keywords “children” and “infants”. The research not only examines the anatomical and functional characteristics of CHD, but also emphasizes its potential impact on infant brain development (52), particularly the mechanism by which perioperative cerebral perfusion changes interfere with the nervous system (53).

Studies have shown that complex CHD can affect brain development through multiple pathways: such as insufficient fetal brain perfusion (seen in Hypoplastic Left Heart Syndrome (HLHS) and Transposition of the Great Arteries (TGA) patients) (54), intraoperative hypoperfusion/hypothermia, and postoperative chronic low cardiac output, all of which can affect gray matter volume growth, myelination, and functional connectivity development. The assessment of these impacts relies heavily on technological advancement. MRI can assess brain volume, white matter development status, delayed gray matter areas, metabolic disorder, or cerebellar development abnormalities at different stages before and after surgery (55, 56).

Empirical studies demonstrate this technology-driven approach (57). used fetal cardiac ultrasound combined with brain MRI and found that HLHS and TGA fetuses already had smaller brain volumes and slower cortical folding in the third trimester of pregnancy (58); compared ultrasound with neonatal MRI and identified delayed volume development in brain regions such as the frontal and parietal lobes in CHD patients, suggesting an early association with postoperative neurodevelopmental disorders.

The understanding of the heart-brain association mechanism in CHD is deepening. Cutting-edge cross-synaptic neural tracing studies have revealed precise neural circuits for “top-down” control and “bottom-up” perception between the heart and the brain (59–61), providing a neurobiological basis for explaining early brain function abnormalities in CHD patients. At the same time, multi-organ imaging techniques of the heart-brain axis can non-invasively provide a complete information chain from cardiac function to brain metabolism (62), opening up new avenues for precise assessment and intervention in the CHD population.

#### 4.1.3 Artificial intelligence and imaging

Artificial intelligence (AI) and deep learning technologies have become the key driving forces for advancing neuro and cardiac image analysis from “image acquisition” to “automatic quantification” and “structural modeling”. Relevant keywords such as “deep learning”, “artificial intelligence”, and “segmentation” have frequently emerged since 2020. Their applications mainly fall into two directions:

In the research of pediatric heart diseases, AI is used to address clinical data challenges. For instance, some studies have applied generative adversarial networks and deep residual networks to synthesize cardiac MRI images, thereby enhancing the training data for complex congenital heart disease models (63); another study designed a segmentation framework based on convolutional neural networks and attention mechanisms, achieving automatic extraction and quantitative analysis of complex ventricular morphology and vascular connections (64).

In the research of pediatric brain diseases, AI is mainly applied to improve analysis efficiency and standardization. The research focuses on the automatic segmentation of neonatal brain structure MRI, combined with template registration to assess the developmental changes in brain tissue volume, replacing the traditional time-consuming manual annotation process (65–67).

It is worth noting that the research on the brain-heart axis is expanding from single organs to multi-organ networks (such as the construction of the “brain-heart-eye axis”) (68, 69), which points the way for AI to handle more complex multi-organ and multi-omics data in the future to reveal deeper biological mechanisms. At the same time, AI technologies (such as federated learning) are also promoting the establishment of multi-center data sharing, standardized research frameworks for diseases of the heart and brain (70–74). In the future, AI is expected to become a key support for connecting clinical screening, prognosis prediction, and basic mechanism research for HBA.

### 4.2 Function & regulation (cluster 2)

#### 4.2.1 Brain-heart functional connectivity mechanism and the value of HRV

Cluster 2 focuses on the regulation of brain function by the autonomic nervous system (ANS), with HRV being a key physiological indicator reflecting the dynamic organization of brain functional networks. Research has shown that HRV is highly coupled with specific brain networks at rest (75, 76); for instance, HRV can modulate the connection strength between the default mode network (DMN) and the prefrontal cortex.

Understanding of the HRV mechanism has advanced from macroscopic correlations to the neural circuit level. Cross-synaptic neural tracing studies have precisely mapped the bidirectional neural circuits between the heart and the brain (61), providing an anatomical basis for the interaction between HRV and the central autonomic network (CAN). Mechanistically, HRV-fMRI causal modeling has demonstrated that HRV fluctuations can predict changes in brain region connectivity patterns (34, 77–79), revealing a feedforward brain-heart regulatory pathway (80). The neural pathways of the heart-brain axis are at the core of HRV research, and their significance has been validated in clinical models such as neurogenic myocardial stunning (81). Additionally, biochemical pathways such as inflammation link stress-related amygdala activity to cardiovascular pathology (82), forming a molecular pathway of “psychological stress - neuroinflammation - cardiovascular damage" (83).

HRV research also examines the impact of individual differences on brain networks. Individuals with high HRV exhibit greater stability in the default mode network and stronger regulation between the limbic system and the prefrontal cortex (84), and are associated with better psychological resilience (85, 86). At the system level, the microstructural integrity of the central autonomic bundle (CAN) is significantly associated with cardiovascular health indicators, directly linking brain structure, autonomic function, and heart health in MRI imaging (87).

#### 4.2.2 Functional network and key brain regions of emotion regulation

Another theme of Cluster 2 is the exploration of the neural mechanisms of emotional disorders such as anxiety and depression. The corresponding keywords (such as “emotion”, “depression”, “stress”, “anterior cingulate cortex”, “medial prefrontal cortex”) reached their peak during the late 2000s to the mid-2010s. Frequently occurring keywords include “anxiety” and “emotion”.

The study found that emotional regulation is closely coupled with autonomic nerve feedback (represented by HRV), and the related brain regions can be located through functional magnetic resonance imaging (fMRI) (88). Individuals with high HRV show stronger functional connections between the amygdala and the medial prefrontal cortex (mPFC) in the resting state, suggesting that they have stronger emotional resilience (84); in the task state, HRV variability can regulate the activation intensity of regions such as the dorsomedial prefrontal cortex, thereby affecting emotional responses after stress (89). These brain regions (such as insula, ACC) are also key nodes for autonomic nerve control and interoception. The correlation of HRV with their activities links peripheral autonomic nerve rhythms to the stability of the central emotional network (90–93).

The understanding of the mechanisms of emotional regulation has deepened to the level of specific pathways. “Cardiac perception”, which is the processing of brain signals related to the heart, is a key regulatory link between the autonomic nerve and emotional experience (81, 94). At the same time, negative emotions can directly affect the heart through the “stress (amygdala) - neuroinflammation - cardiovascular damage” pathway (82, 95), and anxiety disorders are also clearly related to heart events such as myocardial infarction (61, 96, 97). These findings place the emotional-brain function correlation within the framework of the heart-brain axis composed of multiple pathways, deepening the understanding of the co-occurrence of emotional disorders and cardiovascular diseases.

### 4.3 Risk & pathology (cluster 3)

#### 4.3.1 Cardiovascular risk and cognitive impairment

Cluster 3 focuses on the mechanism by which cardiovascular risk factors affect cognitive function in the elderly, with hypertension being a key risk factor. Long-term hypertension damages brain function through multiple pathways: its mechanical pressure can lead to lesions in small cerebral arteries, white matter hyperintensities (WMH), lacunar infarctions, and other structural damage (98, 99); at the same time, the processes triggered by it, such as blood-brain barrier disruption and inflammation, promote Aβ deposition, coexisting with the pathology of Alzheimer's disease (100, 101); the white matter microstructural changes related to hypertension are also an important basis for cognitive decline (102, 103).

At the imaging assessment level, structural MRI is the core tool for detecting brain damage related to hypertension, and can be used to quantify indicators such as WMH load and hippocampal atrophy (104, 105); while positron emission tomography (PET) imaging can track Aβ deposition, and can be used in conjunction with MRI indicators to assess the process of brain degeneration (106).

Vascular cognitive impairment and dementia (VCID) have been confirmed as an important clinical outcome of this type of cardiovascular-cerebral interaction. The latest review systematically explores how the pathological processes connecting the heart and brain jointly affect cardiovascular and nervous systems, and focuses on the pathogenesis of VCID (51).

#### 4.3.2 White matter lesions and cerebral small vessel disease

In the cognitive impairment study of Cluster 3, abnormal white matter structure and cerebral small vessel disease (CSVD) are prominent imaging focal points. The load of white matter hyperintensities (WMH) is significantly correlated with attention, memory, and executive functions, and can predict long-term cognitive decline and the risk of dementia (107, 108); its progression occurs concurrently with cognitive deterioration, supporting its role as a disease process marker of CSVD (109). Cerebral microbleeds (CMB) as another important MRI marker are also associated with cognitive impairment and the risk of dementia (110, 111). Multiple imaging manifestations of CSVD (such as WMH, lacunes) are closely related to the clinical spectrum of vascular cognitive impairment (112), and are the key intersection point of the cardiocerebral axis - heart diseases can lead or exacerbate CSVD through embolism, abnormal cerebral perfusion, and autonomic nerve imbalance (81).

CSVD leads to neural network disorders through mechanisms such as blood-brain barrier disruption, endothelial dysfunction, chronic inflammation, and white matter fiber damage (113, 114). Structural MRI shows that the load of CSVD is not only directly associated with cognitive decline, but also mediates its effects through hippocampal subregion volume reduction and white matter microstructure damage (115). The understanding of its mechanism has gone beyond the vascular itself, for example, cardiac pathology can affect the brain through the “stress (amygdala) - neuroinflammation - vascular damage” pathway (82), forming a cascade reaction connecting psychological stress, heart risk, and the progression of CSVD.

At the imaging technology level, T2-FLAIR is the standard method for detecting WMH, while FLAIR and DTI indicators can complementarily predict the progression of WMH and the risk of white matter degeneration (116, 117). Future trends tend to adopt multi-organ imaging strategies to simultaneously assess the interaction of organs such as the heart and brain, in order to systematically reveal the mechanism of the cardiocerebral axis (62).

This module is highly consistent with keywords such as “white matter lesions” (emergent period 1997–2012) and “cerebral small vessel disease” (2023–2025), as well as high-frequency words “dementia”, “alzheimer's disease”, and “white matter”.

### 4.4 Hemodynamics & perfusion (cluster 4)

#### 4.4.1 Cerebral hemodynamics and perfusion

The core of Cluster 4's research is the brain perfusion function and the regulatory mechanism of hemodynamics, focusing on how the cerebral vascular system adapts to cardiac output through blood flow velocity, perfusion stability, and vascular responsiveness.

Cerebral blood flow (CBF) and cerebral vascular reactivity (CVR) are two key imaging parameters for evaluating brain perfusion, mainly measured using MRI techniques such as arterial spin labeling (ASL). The study found that CBF shows a significant downward trend from normal cognition to mild cognitive impairment (MCI) and to Alzheimer's disease (AD), and the reduction in perfusion in specific brain regions is correlated with the decline in cognitive scores (118, 119). CVR reflects the vascular response to stimuli such as CO₂, and in MCI individuals, the CVR in multiple brain regions is significantly reduced, and its level is related to executive function, memory, and other cognitive performances (120, 121).

In recent years, wall shear stress (WSS), the mechanical force generated by blood flow on the vascular endothelium, has received attention for its regulatory role in the homeostasis of the blood-brain barrier (BBB). Studies have shown that physiological WSS promotes the expression of endothelial tight junction proteins to maintain BBB integrity, while abnormal WSS can damage BBB function through pathways such as disrupting tight junctions and activating inflammation (122, 123).

#### 4.4.2 Multimodal imaging in blood flow assessment

The integration of multimodal imaging techniques is the key to promoting the development of cerebral blood flow research. To overcome the limitations of a single technique, researchers often combine MRI, PET, and transcranial Doppler (TCD) to achieve multi-angle assessment of cerebral perfusion.

Within the family of MRI techniques, different MRI sequences serve complementary purposes: Phase-Contrast MRI (PC-MRI) can quantitatively measure the total cerebral blood flow and the flow of supplying arteries, and provides a reference for arterial spin labeling (ASL) (124); ASL/pCASL, as a key perfusion assessment method, can non-invasively and absolutely quantify cerebral blood flow (125); at the same time, multi-time-point ASL can more accurately measure perfusion kinetic parameters (126); and Blood Oxygen Level-Dependent Functional MRI (BOLD-fMRI) provides important information for studying the coupling between brain function and blood flow (127). Additionally, in the research around MRI and the heart-brain axis, other imaging techniques are often important supplements: TCD can non-invasively and dynamically assess cerebral vascular reactivity, and has potential in the detection of early cognitive impairment (128); PET can quantitatively assess brain metabolism, and is particularly useful for revealing the pathological mechanisms of neurodegenerative diseases (129).

Currently, imaging techniques are developing towards a deep integration centered on MRI. Hybrid equipment such as positron emission tomography/magnetic resonance imaging can simultaneously assess the anatomical and metabolic changes of organs such as the heart and brain, providing a unique perspective for revealing the interaction mechanism between the heart and brain (62).

These patterns are consistent with the temporal evolution from PET/MRI-based perfusion assessment to TCD-related vascular monitoring and more recent MRI segmentation and *in-vivo* imaging approaches.

## 5 Summary and perspectives

This study systematically revealed the knowledge structure and development trajectory of the field of heart-brain interaction over the past four decades by bibliometric analysis. Based on the keyword co-occurrence analysis in VOSviewer, the literature was classified into four main clusters, which respectively describe the heart-brain interaction mechanism from different aspects: (1) Technology & Development highlights the role of MRI in assessing ischemic encephalopathy and brain development in children with congenital heart disease, and incorporates advanced image analysis approaches such as artificial intelligence; (2) Function & Regulation delineates the neural mechanisms of heart–brain functional connectivity and emotion regulation, with heart rate variability as a central physiological marker; (3) Risk & Pathology elaborates on how cardiovascular risk factors shape late-life cognitive impairment and on the links between cerebral small vessel disease and white matter lesions; (4) Hemodynamics & Perfusion concentrates on the evaluation of cerebral hemodynamic parameters and the use of multimodal imaging techniques to characterize brain perfusion and vascular reactivity. The main clusters and keyword structures derived from the Web of Science dataset showed a high degree of concordance with those obtained from a PubMed validation dataset, supporting the robustness of the mapped research themes despite database-specific indexing differences. Future directions in heart–brain axis (HBA) research include a growing emphasis on multi-organ axis modeling and the deeper integration of multimodal imaging and artificial intelligence. By highlighting key directions and providing a knowledge map, this study may help researchers identify frontier topics, assist clinicians in understanding MRI-based heart–brain evidence, and support future patient-oriented studies and healthcare planning on mechanisms, early risk identification, long-term monitoring, and multidisciplinary management.

This study has several limitations. First, it is constrained by the coverage and indexing policies of WoS and PubMed, as well as by its intentional focus on MRI-related literature. Therefore, the findings should be interpreted as reflecting MRI-centered heart–brain axis research rather than the entire neuroimaging or cardiovascular imaging landscape, and relevant CT/CT perfusion, EEG, PET-only, vascular ultrasound/TCD, or broader multimodal cardiovascular imaging studies may not have been fully captured. In addition, topic-based bibliometric searches may inevitably include noise records, because some publications retrieved by keyword matching may be only marginally related to the conceptual scope of MRI-centered heart–brain axis research (130). Bibliometric results are also influenced by search-term selection, keyword standardization, database indexing, citation behaviors, and interpretive cluster labeling; thus, citation counts, TLS, and burst strength should be viewed as descriptive indicators of scholarly attention rather than direct measures of scientific quality or clinical importance (131). Future work could integrate additional databases, harmonized thesaurus mapping, all-modality search strategies, and sensitivity analyses excluding self-citations.

## Data Availability

The original contributions presented in the study are included in the article/Supplementary Material, further inquiries can be directed to the corresponding authors.
