# Supplementary material for: Research hotspots and trend of the heart-brain axis by MRI: a bibliometric analysis
Source: Front Cardiovasc Med. 2026 Jul 7;13:1825341. doi: 10.3389/fcvm.2026.1825341 (PMC13385264; doi:10.3389/fcvm.2026.1825341)
Supplement: Supplementary file 1 [file Datasheet1.docx]

**search query:**

**TS = ( "congenital heart disease" OR "CHD" OR "cyanotic heart disease" OR "cardiac" OR "heart" OR "cardiopulmonary bypass" OR "perfusion" OR "hypoxia" OR "hemodynamics" OR "oxygen saturation" )**

**AND**

**TS = ( "brain" OR "cerebral" OR "cognition" OR "neurodevelopment" OR "neurodevelopmental" OR "neurological" OR "neuro " OR "executive function" OR "intelligence" OR "brain connectivity" OR "functional connectivity" OR "default mode network" OR "resting-state" )**

**AND**

**TS = ( "MRI" OR "magnetic resonance imaging" OR "fMRI" OR "functional MRI" OR "functional magnetic resonance imaging" OR "neuroimaging" OR "diffusion tensor imaging" OR "DTI" OR "brain mapping" )**

**NOT**

**TS = ( "dce-mri" OR "dsc-mri" OR diffusion OR "diffusion mri" OR "diffusion magnetic resonance imaging" OR "diffusion-weighted imaging" OR "diffusion weighted imaging" OR dti OR dwi OR perfusion OR "perfusion imaging" OR "perfusion mri" OR "perfusion magnetic resonance imaging" OR "perfusion-weighted imaging" OR "contrast-enhanced mri" OR "contrast-enhanced mr" OR "contrast enhancement" OR "mr perfusion" OR "mr spectroscopy" OR "magnetic resonance spectroscopy" OR "magnetic-resonance-spectroscopy" OR spectroscopy OR tractography OR "sw-mri" OR "susceptibility-weighted imaging" OR gadolinium OR gd-dtpa OR "contrast agent" OR "contrast agents" OR tracer OR "tracer bolus passages" OR "relaxation-times" OR relaxation OR "singular-value decomposition" OR coefficient OR delay OR dispersion OR pseudoprogression OR "response assessment" OR resection OR "radiation necrosis" OR "radiation-therapy" OR radiotherapy OR radiosurgery OR "stereotactic radiosurgery" OR survival OR progression OR recurrence OR predict OR glioma OR gliomas OR glioblastoma OR "glioblastoma-multiforme" OR "malignant glioma" OR "malignant gliomas" OR "brain tumor" OR "brain tumors" OR "brain-tumor" OR "brain-tumors" OR "brain metastasis" OR "brain metastases" OR metastasis OR metastases OR meningioma OR lymphoma OR "breast-cancer" OR "prostate-cancer" OR carcinoma OR cancer OR "central nervous system" OR "cns lymphoma" OR "multiple-sclerosis" OR "high-grade glioma" )**

****Supplementary Figure.** Author-keyword-based co-occurrence network of MRI-related heart–brain axis research. The analysis was conducted using author keywords exported from WoSCC. A minimum occurrence threshold of 20 was applied; among 12,218 author keywords, 146 met the threshold and were included in the network. Node size indicates keyword occurrence, link thickness indicates co-occurrence strength, and colors represent algorithm-derived clusters.**

**
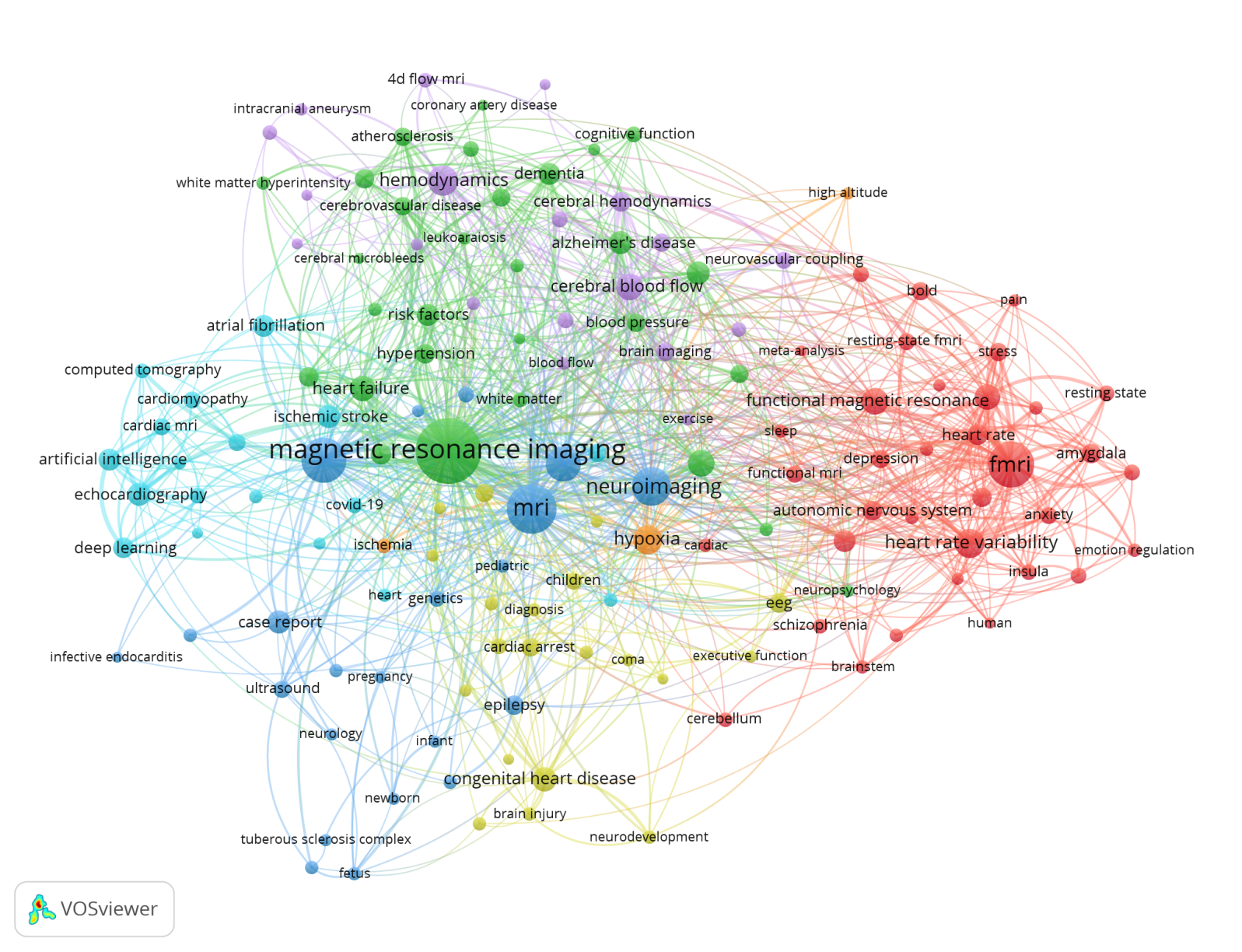
**

****Supplementary Table S1.** Main parameters and preprocessing procedures used in bibliometric analyses.**

| ****Analysis**** | ****Software**** | ****Unit of analysis / data**** | ****Threshold / selection**** | ****Normalization / counting**** | ****Clustering / visualization settings**** | ****Notes**** |
| --- | --- | --- | --- | --- | --- | --- |
| **Main keyword co-occurrence** | **VOSviewer 1.6.20** | **WoSCC all keywords** | **Occurrence ≥50** | **Association strength; full counting** | **Default VOSviewer layout and clustering settings; resolution = 1.00; minimum cluster size = 1; merge small clusters enabled** | **Used for the main keyword clusters** |
| **Supplementary author keywords analysis** | **VOSviewer 1.6.20** | **WoSCC author keywords** | **Occurrence ≥20; 146 of 12,218 keywords included** | **Association strength; full counting** | **Default VOSviewer layout and clustering settings; resolution = 1.00; minimum cluster size = 1; merge small clusters enabled** | **Supplementary keyword-source validation** |
| **Co-authorship: authors** | **VOSviewer 1.6.20** | **Authors** | **Publications ≥5; 495 authors included** | **Association strength; full counting** | **Default VOSviewer layout and clustering settings** | **Author collaboration network** |
| **Co-authorship: organizations** | **VOSviewer 1.6.20** | **Organizations** | **Publications ≥10; 350 organizations included** | **Association strength; full counting** | **Default VOSviewer layout and clustering settings** | **Institutional collaboration network** |
| **Co-authorship: countries/regions** | **VOSviewer 1.6.20 / SCImago Graphica 1.0.51.0** | **Countries/regions** | **Publications ≥5; 65 countries/regions included** | **Association strength; full counting** | **Default VOSviewer layout; geographic visualization generated using SCImago Graphica** | **International collaboration and geographic distribution** |
| **Co-citation: cited references** | **VOSviewer 1.6.20** | **Cited references** | **Citations ≥20** | **Association strength; full counting** | **Default VOSviewer layout and clustering settings** | **Used to map the intellectual base of the field** |
| **Co-citation: cited sources** | **VOSviewer 1.6.20** | **Cited sources** | **Citations ≥50** | **Association strength; full counting** | **Default VOSviewer layout and clustering settings** | **Used to identify core journals/sources** |
| **Burst analysis** | **CiteSpace 6.4.R1** | **Keywords and cited references** | **Top 30 burst keywords and references; time span 1984–2025** | **Default CiteSpace burst detection settings** | **Default CiteSpace visualization settings** | **Used to identify temporal research frontiers** |

****Note:** Before analysis, keywords were manually checked for capitalization differences, spelling variants, abbreviations, duplicate expressions, and semantically overlapping terms where appropriate. No automated stemming or lemmatization algorithm was applied. Cluster labels were interpreted based on high-frequency keywords, TLS values, and representative records within each cluster.**

****Supplementary Table S2.** Representative high-TLS keywords in the four major clusters of the WoSCC keyword co-occurrence network.**

| **Cluster** | **Cluster label** | **Representative high-TLS keyword** | **Occurrences** | **TLS** |
| --- | --- | --- | --- | --- |
| 1 | Technology & Development | MRI | 818 | 2799 |
| 2 | Function & Regulation | functional connectivity | 270 | 1224 |
| 3 | Risk & Pathology | dementia | 270 | 1425 |
| 4 | Hemodynamics & Perfusion | hemodynamics | 286 | 1036 |
